# Higher Sun Exposure in the First Trimester Is Associated With Reduced Preterm Birth; A Scottish Population Cohort Study Using Linked Maternity and Meteorological Records

**DOI:** 10.3389/frph.2021.674245

**Published:** 2021-07-09

**Authors:** Lauren Megaw, Tom Clemens, Konstantinos Daras, Richard B. Weller, Chris Dibben, Sarah Jane Stock

**Affiliations:** ^1^Tommy's Centre for Maternal and Fetal Health, Medical Research Council Centre for Reproductive Health, University of Edinburgh Queen's Medical Research Institute, Edinburgh, United Kingdom; ^2^School of Women and Infants Health, University of Western Australia, Perth, WA, Australia; ^3^School of Geosciences, University of Edinburgh, Edinburgh, United Kingdom; ^4^Institute of Population Health Sciences, University of Liverpool, Liverpool, United Kingdom; ^5^Centre for Inflammation Research, University of Edinburgh Queen's Medical Research Institute, Edinburgh, United Kingdom; ^6^Usher Institute, University of Edinburgh, Edinburgh, United Kingdom

**Keywords:** sunlight, preterm birth, ultraviolet radiation, pregnancy, retrospective cohort, data linkage

## Abstract

**Background:** Preterm birth (birth at <37 weeks gestation) is the leading cause of death in children under 5-years-old, and prevention is a global public health issue. Seasonal patterns of preterm birth have been reported, but factors underlying this have been poorly described. Sun exposure is an important environmental variable that has risks and benefits for human health, but the effects of sun exposure on pregnancy duration and preterm birth are unknown.

**Objectives:** To determine the association between available sun exposure and preterm birth.

**Methods:** We performed a population-based data-linkage study of 556,376 singleton births (in 397,370 mothers) at or after 24 weeks gestation, in Scotland between 2000 and 2010. Maternity records were linked to available sun exposure from meteorological records, by postcode. Logistic regression analysis was used to explore the relationship between available sunshine and preterm birth at <37 weeks gestation. Exploratory analyses included a subgroup analysis of spontaneous and indicated preterm births and a sibling analysis in sib pairs discordant for preterm birth.

**Results:** The rate of preterm birth was 6% (32,958/553,791 live births). Increased available sun exposure in the first trimester of pregnancy was associated with a reduced risk of preterm birth, with evidence of a dose-response. Compared with the lowest quartile of sun exposure, the highest quartile of sun exposure was associated with a reduced odds ratio (OR) of preterm birth of 0.90 (95% Confidence Interval (CI) 0.88–0.94 *p* < 0.01) on univariable analysis and OR of 0.91 (95% CI 0.87, 0.93 *p* < 0.01) after adjustment for second trimester sunlight exposure, parity, maternal age, smoking status, and deprivation category. No association was seen between preterm birth and second trimester available sun exposure or combined first and second trimester exposure. Similar patterns were seen on sibling analysis and within both the indicated and spontaneous preterm subgroups.

**Discussion:** Available sun exposure in the first trimester of pregnancy is associated with a protective effect on preterm birth <37 weeks gestation. This opens up new mechanisms, and potential therapeutic pathways, for preterm birth prevention.

## Introduction

Preterm birth (birth at <37 weeks gestation) is a leading cause of neonatal morbidity and mortality, and deaths in children under 5-years-old worldwide (1). The contribution of environmental factors to preterm birth is not well-studied; (2) however, understanding the impact of the natural environment on pregnancy may present novel pathways for intervention.

Sunlight is a component of the natural environment that is necessary for human health (3). Vitamin D production, nitric oxide production, and activity of the immune system are all influenced directly by sunlight with implications for disease manifestation (4). These pathways are central to the establishment and maintenance of pregnancy, with the early pregnancy state establishing risk for later outcomes (5, 6). However, sun exposure in pregnancy remains mainly incidental and unconsidered. Although there have been relatively few studies, a systematic review of sun exposure and pregnancy outcomes found associations with fetal growth restriction, blood pressure, and preterm birth rates (7, 8), with higher first trimester sunlight correlating with higher fetal birth weights and less hypertensive complications in the third trimester (8). The postulated mechanisms were related to vitamin D generation by sun exposure, deficiency of which in pregnancy is associated with low birth weight, preterm birth, and hypertensive complications of pregnancy (9).

Only one US-based study has explored preterm birth rates and sunlight exposure; however, this study did not address whether ultraviolet (UV) light exposure influenced preterm birth or low birth weight but aimed to assess whether variation in UV light-induced vitamin D synthesis might contribute to racial disparities in birth outcomes in the US, using statewide estimates. To specifically examine the effects of available sunlight on preterm birth requires consideration of exposure periods and individual level adjustment of other maternal data. Using high granularity environmental data applied to an individual pregnancy allows modeling of overall risk related to sun availability and modeling of exposure periods. As latitude increases, the variation offered by larger alterations in the length of day over the calendar year offers a natural experiment in which to examine the effects of available sun exposure. Scotland has high-quality maternity data, and high latitude with variability in sunshine both across and between years, making it an ideal place to study the effects of available sunshine on pregnancy. The objective of this population cohort study was to determine whether there is an association between available sunlight and preterm birth by linking geographically mapped sunlight data to pregnancy and birth records.

## Methods

The study was approved by the privacy advisory service for National Services Scotland approval number PAC91/147. Data available for analysis were pseudo-anonymized and analyzed within a trusted research environment (the NHS Scotland Safe Haven). Findings are reported in accordance with the RECORD checklist for observational studies using routinely collected health data (10).

### Study Population

We used the Scottish Morbidity Record 02 (SMR02) which records information of all women admitted to Scottish maternity hospitals (11). It contains information on maternal and infant characteristics, clinical management, and obstetric complications (11). During the period studied, the SMR02 did not include homebirths however these are <2% of all Scottish births. Regular detailed quality assurance of the SMR02 occurs, and the most relevant to this dataset is the 2008–9 report which confirmed the completeness (>90%) and accuracy of the fields used in this study (11).

### Inclusion and Exclusion Criteria

We extracted data from SMR02 record on all liveborn infants born in Scotland between Jan 1, 2000, and Dec 31, 2010, inclusive. We restricted the analysis to singleton births at or beyond 24 weeks gestation as a feature of the SMR02 database (11). We excluded births with congenital anomalies. We also excluded cases based on *a priori* thresholds of plausibility. Infants were excluded if the gestational length was more than 46 weeks, birth weight was >7,000 g or <350 g, and maternal age was <13 years. Finally, we excluded women with missing gestation at birth, parity, smoking status, or who we could not link with available sunshine exposure.

### Definitions

Preterm birth was considered as a categorical variable, defined as birth before 37 weeks gestation. Ultrasound is routinely used in the first trimester of pregnancy in Scotland to determine gestational age (12). We imputed the date of conception from the date of delivery, minus gestational age at delivery plus 2 weeks. Trimester 1, we defined as the first 12 weeks of pregnancy from conception and trimester 2, as 13–28 weeks. In the sensitivity analysis, we also used completed gestational weeks as a continuous variable.

The mean daily sunlight exposure was calculated for each trimester and during the whole pregnancy for each woman. We did not use data on available sun exposure during the third trimester of pregnancy, because most preterm births occur during the third trimester, which complicates the exposure duration of available sunlight during this period. To represent cumulative sunlight exposure, a value was calculated for the mean of trimester 1 and 2 called “average trimester 1 and 2” exposure.

We defined “spontaneous” preterm births as women who gave birth <37 weeks gestation, who did not have an elective cesarean section or an induction of labor. We defined “provider initiated” preterm births as women who gave birth <37 weeks gestation, who had an elective cesarean section or an induction of labor.

Postcodes of residence, which are highly geographically specific, were used to link to meteorological data in 5 ×5 km grid squares, generated from two sources, the UK meteorological (Met) office (13) and EUMETSAT (14). The Met office is weather observation and prediction service of the UK funded under the Department for Business Innovation and Skills (13). The Met office data is freely available including monthly average sunlight hours over a grid of Scotland with each grid value referencing a 5 ×5 km surface area of Scotland. EUMETSAT includes geostational meteorological satellites covering Europe. In EUMETSAT, freely available data include the Meteosat series of satellites, which provide daily values for surface solar insolation at a spatial resolution of 1° of latitude and longitude. Met office and Meteosat data were combined to provide mean sunlight hours a day for each 5 ×5 km grid square across Scotland, for every day of the exposure period (from January 1, 1999 to December 31, 2010).

### Potential Confounders

We took a first-principles approach to identifying confounders of the sunlight and preterm birth relationship utilizing directional acyclic graphs (DAGs) to determine the primary modeling approach (Supplementary Figure 1). Available sunlight and pregnancy outcome are at low risk of confounding using this approach, as very little is deterministically associated with available sunlight. We considered adjustment for a season of conception as available sunlight in the northern latitudes is highly correlated with the season, and the season of conception has been variably associated with preterm birth. However, season likely acts as a proxy for seasonal reproductive behavior, variation in temperature, the burden of winter influenza, seasonal changes in pollen counts, and particulate air pollution all of which have the potential to be mediated by available sunlight. We also note the approach recommended by Weinberg et al. (15) who demonstrated that if measures of social confounding are available, preferentially modeling these instead of utilizing season as a surrogate is more analytically rational (12). As such the primary logistic regression model did not include season of conception but did include sociodemographic variables including maternal age at birth (categorized as ≤ 18, 19–29, 30–34, 35–39, ≥40 years of age), smoking in pregnancy (yes/no), parity, and socioeconomic deprivation [derived from Scottish Index of Multiple Deprivation (SIMD) Quintiles, allocated by postcode (16) (model 1)]. We did include season of conception in an additional model (model 2) recognizing the potential for over-adjustment in this model. We defined the season of conception meteorologically with December–February as winter, March–May as spring, June–August as summer, and September–November as autumn (16).

For the “trimester 1” and “trimester 2” exposure models, we adjusted for the alternative trimester of exposure, available sunlight exposure in the preceding trimester (for second trimester exposure) or subsequent trimester (for first trimester exposure) for both model 1 and model 2. The “average trimester 1 and 2” exposure was not adjusted for any other exposure variable.

### Statistical Analysis

For descriptive statistics of continuous variables, we used mean and SD for normally distributed data, and median and interquartile range (IQR) for non-parametric data. Categorical data were presented as percentages with 95% Confidence Intervals (CI). We modeled odds ratios (OR) of preterm birth using logistic regression, before and after adjustment for confounders. *P* <0·05 were considered statistically significant.

### Sensitivity Analysis

We undertook the primary analysis described above and also controlled for within-mother effects using conditional fixed effects regression by using the national identifier [Community Healthcare Index (CHI)] to identify mothers within the dataset. We also modeled available sun exposure with the gestational age at delivery in completed weeks as a continuous variable using linear regression with univariate and multivariate models as described for the primary analysis.

We did a sibling analysis as a sensitivity analysis to explore the effect of any potential residual confounding. We identified mothers who had experience both a term birth and a preterm birth and compared the sun exposure between these pregnancies using conditional logistic regression with mother-level fixed effects. In the sibling analysis, we compared available sunlight exposure during pregnancy in sib-pairs discordant for preterm birth. We analyzed the whole group, as well-separate analyses to adjust for the season of conception and maternal age, smoking status, SIMD category, and parity.

### Subgroup Analysis

To explore potential underlying mechanisms, we performed a subgroup analysis of spontaneous preterm births of <37 weeks and indicated preterm births of <37 weeks.

All analyses were done with Stata (version 14).

## Results

**Figure 1:**
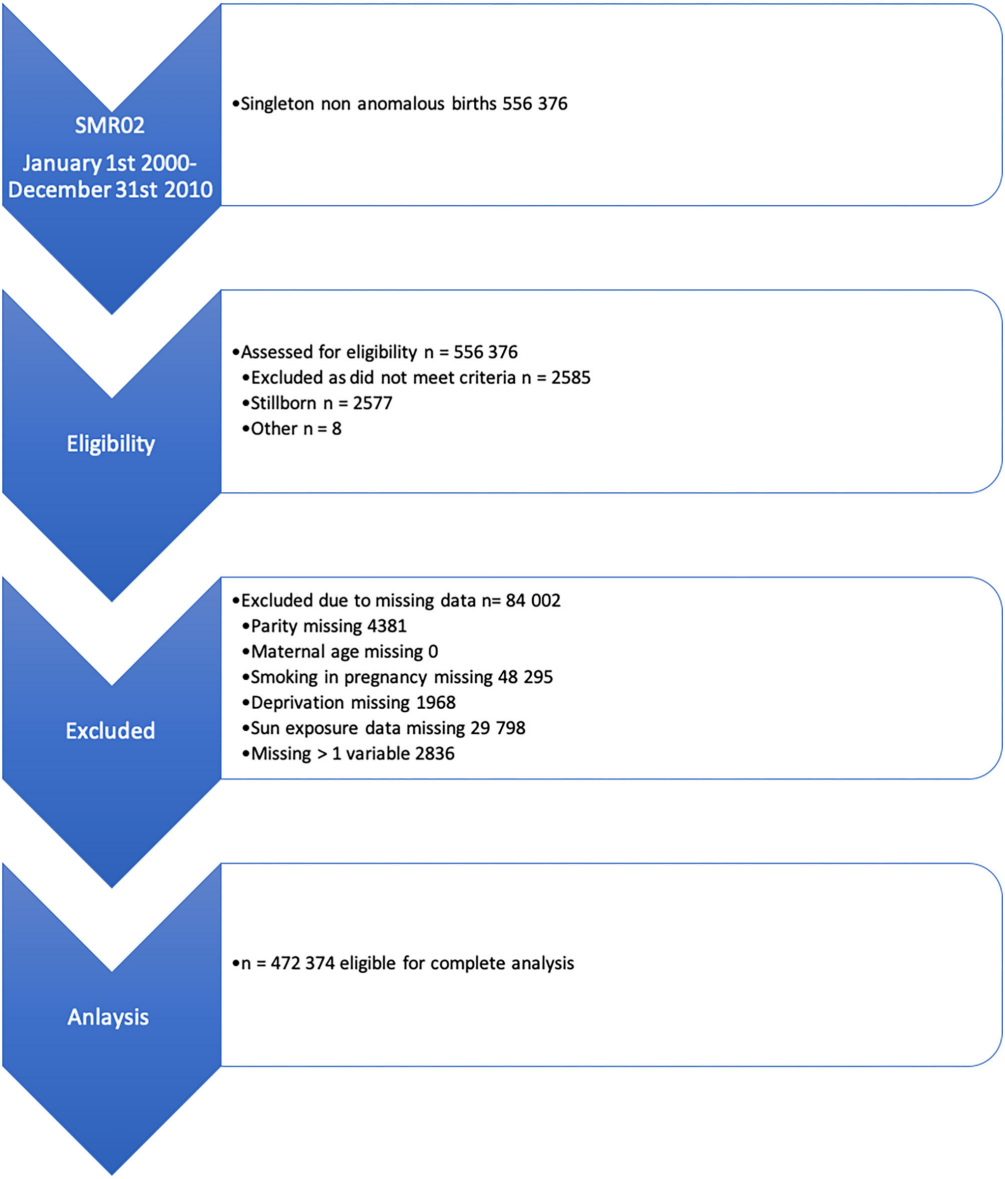
Inclusion and exclusion flow chart for the study population.

Between Jan 1, 2000, and Dec 31, 2010, there were 553,791 live singleton births recorded in Scotland. Of these births, we excluded 81,417 (Figure 1). The analysis cohort consisted of 472,374 births to 395,588 mothers. Of these births, 32,958 (6.0%) were preterm. The characteristics of the cohort are described in Table 1, stratified by quartile of available sun exposure in trimester 1.

**Table 1:** Maternal characteristics of all non-anomalous singleton births occurring in Scotland 2000–2010 by quartile of available sun hours in the first trimester of pregnancy.

	**Q1 (least)**	**Q2**	**Q3**	**Q4 (most)**	**Overall**
Live singleton births (%)	131,032 (25)	131, 001 (24.9)	131,069 (25)	131,073 (25)	524,175 (100)
Mothers, *n*					395,588
Maternal age, mean (SD), years	28.8 (6.1)	28.9 (6.1)	28.9 (6.1)	28.9 (6.1)	28.8 (6.1)
Primiparity, *n* (%)	59,994 (46.1)	60,861 (46.8)	59,967 (46.1)	61,136 (47.1)	241,958 (46.5)
Smoker pregnancy, *n* (%)	29,158 (22.2)	27,948 (21.3)	27,792 (21.2)	27,626 (21.1)	112,524 (21.5)
Missing (%)	12,919 (9.9)	11,872 (9.1)	11,767 (9.0)	10,469 (8.1)	47,027 (9.0)
Deprivation category (SIMD) *n* (%)					
1 (least)	21,534 (16.4)	23,406 (17.9)	23,795(18.2)	23,873 (18.2)	92,608 (17.7)
2	23,231 (17.7)	23,908 (18.3)	23,795 (18.1)	23,846(18.2)	94,780 (18.1)
3	24,878 (19.0)	24,370(18.6)	24,467 (18.7)	24,171 (18.4)	97,886 (18.7)
4	27,191 (20.8)	26,517 (20.2)	26,459 (20.2)	26,983 (20.6)	107,240 (20.5)
5 (most)	34,072 (26.0)	32,642 (23.6)	32,922 (25.1)	32,053 (24.5)	131,689 (25.1)
Missing *n* (%)	126 (0.1)	158 (0.1)	175 (0.1)	147 (0.1)	606 (0.1)
Birthweight (mean, SD) g	3392.0 (581.3)	3404.2 (579.2)	3411.3 (577.8)	3416.2 (572.8)	3400.2 (586.7)
Gestation at birth (median, IQR) weeks	40 (2)	40 (2)	40 (2)	40 (2)	40 (2)
Preterm birth <37 weeks *n* (% of births)	8,150 (6.2)	7,773 (5.9)	7,595 (5.8)	7,412 (5.7)	30,930 (5.9)
Spontaneous preterm birth <37 weeks *n* (% of preterm births)	6,959 (85.4)	6,680 (85.9)	6,538 (86.0)	6,372 (86.0)	26,549 (85.8)
Indicated preterm birth <37 weeks *n* (% of preterm births)	1,168 (14.3)	1,077 (13.9)	1,017 (13.4)	1,011 (13.6)	4,273 (13.8)
Sibling pairs discordant for preterm birth, *n*	-	-	-	-	9, 054

*Q, Quartile of exposure; Q1, least exposed; Q4, most exposed*.

Over the study period, the mean sunlight exposure hours per day ranged from 1.59 in winter months to 6.71 h in summer months (Supplementary Table 1). The annual distribution was unimodal with a summer peak. Variation in exposure between years was evident primarily in differences in the available summer sunlight. An indication of spatial variation is given in Supplementary Figure 2, with a map showing variation in average trimester 1 exposure for births delivered in 2001 across 5 ×5 km areas in Scotland.

### Relationship Between Available Sun Exposure and Preterm Birth

Available sun exposure in trimester 1 of pregnancy was inversely associated with preterm birth in univariable and multivariable models with evidence of a dose-dependent effect (Table 2). Compared with the lowest quartile of exposure, the highest quartile of exposure was associated with a reduced OR of preterm birth of 0.90 (95% CI 0.88–0.94 *p* < 0.01) on univariable analysis with a small attenuation of effect size in the adjusted models but a persistent significant dose-dependent protective effect [model 1 OR 0.91 (95% CI 0.87, 0.93 *p* < 0.01)] (Table 2). However, available sun exposure in trimester 2 was not associated with preterm birth (OR 1.02 95% CI 0.99–1.06, *p* 0.12). The average trimester 1 and 2 exposure had a weakened but similar effect to the trimester 1 exposure, confirming the persistence of the trimester 1 effect regardless of trimester 2 with the highest quartile of exposure associated with a reduced OR of preterm birth of 0.95 (95% CI 0.92–0.99, *p* 0.01) and in the adjusted model 1 OR 0.96 (95% CI 0.93–1.00 *p* 0.04). The results were unchanged controlling for within mother effects (Supplementary Table 2). Using linear regression for gestational length, increasing available sun exposure was associated with increasing gestational length with the highest exposure quartile of exposure β Coefficient 0.07 (95% CI 0.05–0.08, *p* < 0.01) (Supplementary Table 3).

**Table 2:** Association between mean quartile of sun hours available per day in trimester 1, trimester 2 and the combined average exposure over trimester 1 and 2, and preterm birth in Scotland 2000–2010 using logistic regression.

**Available sun exposure**	**Preterm births *n* (%)**	**Univariable OR (95% CI)**	***p***	**Multivariable OR (95% CI) model 1**	***p***	**Multivariable OR (95% CI) model 2**	***p***
**Trimester 1**							
Q1 (lowest)	8,150 (6.2)	Ref		Ref		Ref	
Q2	7,773 (5.9)	0.95 (0.92, 0.98)	<0.01	0.94 (0.91, 0.98)	<0.01	0.98 (0.94, 1.03)	0.47
Q3	7,597 (5.7)	0.93 (0.89, 0.96)	<0.01	0.92 (0.86, 0.95)	<0.01	0.96 (0.92, 1.01)	0.13
Q4 (highest)	7,412 (5.6)	0.90 (0.88, 0.94)	<0.01	0.91 (0.87, 0.93)	<0.01	0.94 (0.89, 0.98)	<0.01
**Trimester 2**							
Q1 (lowest)	7,731 (5.9)	Ref		Ref		Ref	
Q2	7,726 (5.8)	1.01 (0.97, 1.04)	0.72	0.98 (0.95, 1.02)	0.47	0.98 (0.93, 1.02)	0.27
Q3	7,698 (5.9)	1.00 (0.97, 1.04)	0.95	0.97 (0.94, 1.01)	0.25	0.97 (0.92, 1.02)	0.21
Q4 (highest)	7,776 (5.9)	1.02 (0.99, 1.06)	0.19	1.02 (0.99, 1.06)	0.11	1.04 (0.99, 1.10)	0.12
**Average Trimester 1+2**							
Q1 (lowest)		Ref		Ref		Ref	
Q2		1.01(0.98, 1.04)	0.47	1.01(0.98, 1.05)	0.32	1.01(0.98, 1.05)	0.47
Q3		0.94 (0.91, 0.98)	<0.01	0.95 (0.92, 0.98)	0.01	0.96 (0.92, 1.01)	0.10
Q4 (highest)		0.95 (0.92, 0.99)	0.01	0.96 (0.93, 1.0)	0.04	0.98 (0.93, 1.03)	0.53

*Q, Quartile*.

*Model 1 adjusted for alternate trimester exposure, maternal age, smoking status, SIMD category and parity; for exposure “average trimester 1+2” adjusted for maternal age, smoking status, SIMD category and parity*.

*Model 2 adjusted for alternate trimester exposure, season of conception, maternal age, smoking status, SIMD and parity; for exposure “average trimester 1+2” adjusted for season of conception, maternal age, smoking, SIMD category and parity*.

The sibling analysis included 9,054 sibling pairs and showed an inverse relationship between preterm birth and sun exposure in the first trimester similar to the full cohort (Table 3).

**Table 3:** Association between mean sun hours per day available in trimester 1 and 2 and the combined average exposure over trimester 1 and 2, within sibling pairs where one sibling is born term and the other preterm to the same mother within Scotland 2000–2010.

**Available sun exposure**	**Sibling univariable preterm birth OR (95% CI)**	***p***	**Sibling multivariable preterm birth OR (95% CI) Model 1**	***p***	**Sibling multivariable preterm birth OR (95% CI) Model 2**	***p***
Trimester 1	0.98 (0.96, 1.0)	0.02	0.98 (0.96, 1.0)	0.11	0.98 (0.95, 1.01)	0.19
Trimester 2	1.00 (0.98, 1.02)	0.78	1.01 (0.99, 1.03)	0.19	1.04 (1.01, 1.08)	<0.01
Average trimester 1+2	0.98 (0.91, 1.06)	0.74	1.0 (0.97, 1.02)	0.82	1.02 (0.97, 1.06)	0.34

*Model 1 adjusted for alternate trimester exposure, maternal age, smoking status, SIMD category and parity; for exposure “average trimester 1+2” adjusted for maternal age, smoking status, SIMD category and parity*.

*Model 2 adjusted for alternate trimester exposure, season of conception, maternal age, smoking status, SIMD and parity; for exposure “average trimester 1+2” adjusted for season of conception, maternal age, smoking, SIMD category and parity*.

This outcome was seen in the spontaneous and indicated preterm birth analysis with persistent dose-dependent effect sizes for the inverse relationship between available sunlight in trimester 1 and preterm birth (Tables 4, 5).

**Table 4:** Association between mean sun hours per day available in trimester 1 and 2 and the combined average exposure over trimester 1 and 2 and spontaneous onset of preterm labour in Scotland 2000–2010.

**Available sun exposure**	**Spontaneous onset of preterm labour**					
	**OR (95% CI) univariate**	***p***	**Model 1 OR (95% CI)**	***p***	**Model 2 OR (95% CI)**	***p***
**Trimester 1**						
Q1 (lowest)	Ref		Ref		Ref	
Q2	0.95 (0.93, 0.99)	0.02	0.94 (0.91, 0.98)	<0.01	0.98 (0.94, 1.03)	0.57
Q3	0.93 (0.90, 0.97)	<0.01	0.92 (0.88, 0.96)	<0.01	0.97 (0.92, 1.02)	0.27
Q4 (highest)	0.91 (0.88, 0.94)	<0.01	0.91 (0.88, 0.95)	<0.01	0.95 (0.90, 1.00)	0.07
**Trimester 2**						
Q1 (lowest)	Ref		Ref		Ref	
Q2	0.99 (0.95, 1.02)	0.56	0.98 (0.94, 1.02)	0.27	0.97 (0.92, 1.01)	0.15
Q3	1.00 (0.91, 1.03)	0.94	0.99 (0.88, 0.96)	0.50	0.97 (0.92, 1.02)	0.29
Q4 (highest)	1.01 (0.98, 1.04)	0.51	1.03 (0.99, 1.08)	0.06	1.04 (0.98, 1.00)	0.17
**Average Trimester 1+2**						
Q1	Ref		Ref		Ref	
Q2	1.01 (0.98, 1.05)	0.37	1.03 (0.99, 1.07)	0.09	1.03 (0.99, 1.07)	0.12
Q3	0.95 (0.92, 0.99)	<0.01	0.96 (0.92, 1.00)	0.05	0.98 (0.93, 1.03)	0.42
Q4	0.96 (0.92, 0.99)	0.02	0.98 (0.95, 1.02)	0.55	1.02 (0.96, 1.08)	0.56

*Q, Quartile*.

*Model 1 adjusted for alternate trimester exposure, maternal age, smoking status, SIMD category and parity; for exposure “average trimester 1+2” adjusted for maternal age, smoking status, SIMD category and parity*.

*Model 2 adjusted for alternate trimester exposure, season of conception, maternal age, smoking status, SIMD and parity; for exposure “average trimester 1+2” adjusted for season of conception, maternal age, smoking, SIMD category and parity*.

**Table 5:** Association between mean sun hours per day available in trimester 1 and 2 and the combined average exposure over trimester 1 and 2 and indicated preterm births in Scotland 2000–2010.

**Available sun exposure**	**Indicated preterm birth**					
	**OR (95% CI) unviariate**	***p***	**Model 1 OR (95% CI)**	***p***	**Model 2 OR (95% CI)**	***p***
**Trimester 1**						
Q1 (lowest)	Ref		Ref		Ref	
Q2	0.93 (0.87, 0.99)	0.02	0.93 (0.87, 1.01)	0.08	0.95 (0.88, 1.03)	0.30
Q3	0.91 (0.895, 0.97)	<0.01	0.90 (0.84, 0.97)	<0.01	0.92 (0.84, 1.01)	0.09
Q4 (highest)	0.89 (0.83, 0.95)	<0.01	0.89 (0.83, 0.95)	<0.01	0.89 (0.81, 0.98)	0.02
**Trimester 2**						
Q1 (lowest)	Ref		Ref		Ref	
Q2	1.02 (0.95, 1.08)	0.60	0.99 (0.92, 1.07)	0.81	1.00 (0.92, 1.09)	0.99
Q3	0.96 (0.90, 1.03)	0.23	0.95 (0.88, 1.02)	0.15	0.97 (0.8, 1.08)	0.60
Q4 (highest)	0.99 (0.93, 1.06)	0.84	1.00 (0.94, 1.08)	0.90	1.06 (0.95, 1.19)	0.27
**Average Trimester 1+2**						
Q1 (lowest)	Ref		Ref		Ref	
Q2	0.95 (0.89, 1.01)	0.12	0.97 (0.91, 1.04)	0.41	0.97 (0.90, 1.04)	0.33
Q3	0.91 (0.85, 0.97)	<0.01	0.92 (0.86, 0.99)	0.02	0.92 (0.84, 1.01)	0.07
Q4 (highest)	0.88 (0.83, 0.95)	<0.01	0.90 (0.84, 0.96)	<0.01	0.90 (0.81, 0.99)	0.04

*Q, Quartile*.

*Model 1 adjusted for alternate trimester exposure, maternal age, smoking status, SIMD category and parity; for exposure “average trimester 1+2” adjusted for maternal age, smoking status, SIMD category and parity*.

*Model 2 adjusted for alternate trimester exposure, season of conception, maternal age, smoking status, SIMD and parity; for exposure “average trimester 1+2” adjusted for season of conception, maternal age, smoking, SIMD category and parity*.

## Discussion

We found a robust association between available sunlight in the first trimester and a reduction in the risk of preterm birth. This effect appears to be dose dependent and the effect is only minimally attenuated by adjustment of the models. The relationship also persists in the subgroup analyses of the sibling, spontaneous and indicated preterm birth groups supporting the strength of this relationship.

Only one other study has examined sunlight and preterm birth risk. Thayer et al. (17) investigated the role of sunlight in accounting for differences in preterm birth rates between white and non-Hispanic black populations in the US. Their methodology used a statewide average measure of the UV index as the exposure variable and aggregated statewide data and found that as average annual UV increased, the disparity in preterm birth rates between white and non-Hispanic black women increased concluding that in the US the socioeconomic factors co-vary with the UV index and that sunlight availability (which they considered an instrument for photosynthesis of Vitamin D) were not responsible for the race-based disparities in preterm birth. This study methodology strives to refine the limitations of Thayer's study using highly granular environmental data in both space and time, linked at an individual level, alongside a less racially varied study population, which may account for significantly different findings in this study (8). The effect of an annual average UV index alone does not overcome patterning of births related to social disparity and does not contradict this study finding that available early pregnancy sunlight may be protective for preterm birth.

As explored in DAG (Supplementary Figure 1), sunlight availability is an environmental exposure variable that is quite protected from confounding and measurement error bias as this is unlikely to be introduced by satellite data. The main potential confounder is the season of conception which represents a clustering of biological, social, behavioral, and environmental factors rather than a discrete entity. In methodological reproductive work, numbers of conceptions and, therefore, births vary by season which may account for some of the observed seasonal variations in gestational length, utilizing season of conception as we have done rather than a season of birth accommodating this (15, 18). Adjusting for measurable aspects of “season,” such as markers of deprivation and behavior, reduces “seasonal” variation in preterm birth outcomes, whereas in Weinburg's study in Norway (15), adjusting for the season of conception and maternal characteristics ameliorated seasonal variation in gestational length. Curie (18) took a “within mothers” sibling design approach to address seasonal variation in birth outcomes, specifically gestational length and birth weight, and showed that even adjusting within siblings, a May conception (or spring in the northern hemisphere) remained associated with a shorter gestational length and hypothesized that this may be attributable to seasonal influenza.

We prefer the approach that the season of conception is not a discrete entity and adjusting for the season in addition to maternal confounders is over adjustment that hypothetically would then attenuate effect. Our data support this hypothesis, with an increasing reduction in effect size with the addition of season of conception into the model and that the effect remains, even if reduced in size, supports the strength of the relationship between first trimester sunlight and preterm birth.

Season of conception has been previously associated with preterm birth, and in a London cohort, winter birth was associated with a 10% increased risk of preterm birth (19). However, whether this observation was due to a biological pathway or due to potential methodological limitations of the study is unclear. Weinberg et al. (15) demonstrated that seasonal influences on preterm birth are weakened by taking a “fetus at risk” approach. This is because assessment of preterm birth that does not include consideration of the population of fetuses at risk will bias the data to appear as though more preterm births occur at a time when more women are pregnant, i.e., a greater number of fetuses are at risk. Adjusting for the season of conception ameliorates this bias. Weinberg et al. (15) also demonstrated that seasonal effects on preterm birth are stronger in unplanned rather than planned conceptions. Unplanned pregnancy, smoking, low levels of education, and non-married status are risk factors for preterm birth and also bias pregnancy dating by recalled last menstrual period (LMP). Weinberg et al. (15) concluded that if measures of social confounding are available, preferentially modeling these instead of utilizing season as a surrogate is more analytically rational. We have followed this approach within this study.

It is biologically plausible that higher sunlight availability in trimester 1 has downstream effects on the gestational length and, therefore, preterm birth by improving implantation or early placentation. The determination of gestational length is complex and poorly understood, with maternal age, body mass index (BMI), and previous genetic predisposition interacting with intrinsic pregnancy factors (20). The essential component of sunlight, UV radiation, reduces blood pressure potentially by stimulating nitric oxide release and also modulates the immune system (4, 21), these are essential physiological mediators in the process of implantation, early placentation, and thus tolerance of pregnancy (22–24). Subtle deficits in early placentation become apparent in later pregnancy and can manifest as both spontaneous and iatrogenic preterm birth due to the classic obstetric complications of pre-eclampsia and fetal growth restriction (24, 25). These conditions are placentally mediated and are significant contributors to iatrogenic preterm birth in either the fetal or maternal interest but often co-exist with spontaneous onset of preterm labor (24). We observed similar effects in both spontaneous and iatrogenic preterm birth models, suggesting a pathophysiological role for higher available sunlight promoting conditions for more favorable implantation or placentation and thus reducing preterm birth.

This large epidemiological study increases the understanding of the protective effect of early pregnancy sunlight on the gestational length in a high latitude country. As preterm birth remains the leading contributor to neonatal death, understanding environmental influences open novel research pathways to investigate strategies to reduce preterm birth and hence childhood morbidity and mortality.

## Conclusion

In Scotland, higher environmental sunlight availability in the first trimester of pregnancy has significant dose-dependent protective effects on preterm birth that are applicable to the whole singleton pregnancy population. This effect is seen in spontaneous and indicated preterm births, suggesting a likely early pregnancy effect on the maternal vascular and immunological adaptation to pregnancy. This opens novel research pathways to explore both mechanisms and interventions to reduce preterm birth.

## Data Availability Statement

The original contributions presented in the study are included in the article/Supplementary Material, further inquiries can be directed to the corresponding author/s.

## Author Contributions

This original research idea was developed by SS, LM, CD, and RW with project development and analysis by LM and TC. Input by KD regarding environmental exposure and contribution to development of research methods. Final manuscript prepared by LM with contribution from all authors to development. All authors contributed to the article and approved the submitted version.

## Conflict of Interest

The authors declare that the research was conducted in the absence of any commercial or financial relationships that could be construed as a potential conflict of interest.

## Supplementary Material

Supplementary Figure 1 Directional Acyclic Graph for main model. DAG shows the role of potential confounders in the relationship between environmental sunlight availability and gestational length of pregnancy. Adjusted and unadjusted variables are represented by white and blue circles respectively. This model indicates an unbiased pathway from sun availability to the outcome.

Supplementary Figure 2 Example of spatial variation in sun availability. Map shows average trimester 1 sun hours per day for pregnancies delivered in 2001 for each 5 ×5 km square in Scotland.
