# Supplementary material for: Higher Sun Exposure in the First Trimester Is Associated With Reduced Preterm Birth; A Scottish Population Cohort Study Using Linked Maternity and Meteorological Records
Source: Front Reprod Health. 2021 Jul 9;3:674245. doi: 10.3389/frph.2021.674245 (PMC9580751; doi:10.3389/frph.2021.674245)
Supplement: Supplementary file 1 [file Table_1.DOCX]

Supplementary Table 1: Mean sun hours available per day during the whole of pregnancy and each trimester of all singleton pregnancies in Scotland 2000-2010

|  | Sun exposure, mean hrs (SD) |
| --- | --- |
| Pregnancy | 3.71 (0.61) |
| 1^st^ trimester | 3.77 (1.51) |
| 2^nd^ trimester | 3.69 (1.52) |
| 3^rd^ trimester | 3.53 (1.67) |

SD standard deviation

Supplementary Table 2

Association between mean quartile of sun hours available per day in trimester 1, trimester 2 and the combined average exposure over trimester 1 and 2, and preterm birth in Scotland 2000-2010 using logistic regressioncontrolled for within mother effects

| Available sun exposure | Preterm birthsn(%) | Univariable  OR (95% CI) | p | Multivariable, OR (95% CI)  **Model 1** | p | Multivariable OR (95% CI)  **Model 2** | p |
| --- | --- | --- | --- | --- | --- | --- | --- |
| Trimester 1  Q1 (lowest)  Q2  Q3  Q4 (highest) | 8150 (6.2)  7773 (5.9)  7597 (5.7)  7412 (5.6) | Ref  0.95 (0.92, 0.98)  0.93(0.89, 0.96)  0.90 (0.87, 0.94) | <0.01<0.01<0.01 | Ref  0.94 (0.91, 0.98)  0.92 (0.89, 0.95)  0.91 (0.87, 0.94) | <0.01  <0.01  <0.01 | Ref  0.98 (0.94, 1.03)  0.96 (0.91, 1.02)  0.94 (0.88, 0.99) | 0.49  0.18  <0.01 |
| Trimester 2  Q1 (lowest)  Q2  Q3  Q4 (highest) | 7731 (5.9)  7726 (5.8)  7698 (5.9)  7776 (5.9) | Ref  1.01 (0.97, 1.04)  1.00 (0.97, 1.04)  1.02 (0.99, 1.06) | 0.72  0.95  0.19 | Ref  0.99 (0.95, 1.02)  0.98 (0.94, 1.01)  1.03 (0.99, 1.07) | 0.47  0.25  0.11 | Ref  0.98 (0.93, 1.02)  0.97 (0.91, 1.03)  1.04 (0.97, 1.12) | 0.31  0.29  0.22 |
| Average trimester 1+2  Q1 (lowest)  Q2  Q3  Q4 (highest) |  | Ref  1.01 (0.98, 1.04)  0.95 (0.91, 0.98)  0.96 (0.92, 0.99) | 0.47<0.010.01 | Ref  1.02 (0.98, 1.05)  0.95 (0.92, 0.99)  0.96 (0.93, 1.0) | 0.34  <0.01  0.05 | Ref  1.01 (0.98, 1.05)  0.96 (0.91, 1.02)  0.98 (0.93, 1.05) | 0.47  0.18  0.63 |

Q= Quartile

Model 1 adjusted for alternate trimester exposure, maternal age, smoking status, SIMD category and parity; for exposure ‘average trimester 1+2’ adjusted for maternal age, smoking status, SIMD category and parity

Model 2 adjusted for alternate trimester exposure, season of conception, maternal age, smoking status, SIMD and parity; for exposure ‘average trimester 1+2’ adjusted for season of conception, maternal age, smoking, SIMD category and parity

Supplementary Table 3

Association between mean quartile of sun hours available per day in trimester 1, trimester 2 and the combined average exposure over trimester 1 and 2, and gestational length in weeks in Scotland 2000-2010 using linear regression controlled for within mother effects

| Available sun exposure | Preterm birthsn(%) | Univariable  β-Coef  (95% CI) | p | Multivariable,  β-Coef (95% CI)  **Model 1** | p |
| --- | --- | --- | --- | --- | --- |
| Trimester 1  Q1 (lowest)  Q2  Q3  Q4 (highest) | 8150 (6.2)  7773 (5.9)  7597 (5.7)  7412 (5.6) | Ref  0.03 (0.01, 0.04)  0.05 (0.04, 0.07)  0.07 (0.05, 0.08) | <0.01  <0.01  <0.01 | Ref  0.02 (-0.004, 0.04)  0.04 (0.01, 0.06)  0.04 (0.02, 0.07) | 0.12  <0.01  <0.01 |
| Trimester 2  Q1 (lowest)  Q2  Q3  Q4 (highest) | 7731 (5.9)  7726 (5.8)  7698 (5.9)  7776 (5.9) | Ref  0.00 (-0.01, 0.01)  -0.003(-0.02, 0.01)  -0.01(-0.03, -0.00) | 0.97  0.71  0.05 | Ref  0.02 (0.001, 0.04)  0.02 (-0.003, 0.05)  -0.01(-0.04, 0.02) | 0.04  0.08  0.67 |
| Average trimester 1+2  Q1 (lowest)  Q2  Q3  Q4 (highest) |  | Ref  0.01 (-0.01, 0.02)  0.04 (0.02, 0.05)  0.03 (0.02, 0.05) | 0.45  <0.01  <0.01 | Ref  0.01 (-0.01,0.02)  0.03 (0.00, 0.05)  0.02 (-0.01,0.05) | 0.34  0.02  0.20 |

Q= Quartile

Model 1 adjusted for alternate trimester exposure and season of conception; for exposure ‘average trimester 1+2’ adjusted for season of conception
