# Supplementary figures and images for: Higher Sun Exposure in the First Trimester Is Associated With Reduced Preterm Birth; A Scottish Population Cohort Study Using Linked Maternity and Meteorological Records
Source: Front Reprod Health. 2021 Jul 9;3:674245. doi: 10.3389/frph.2021.674245 (PMC9580751; doi:10.3389/frph.2021.674245)

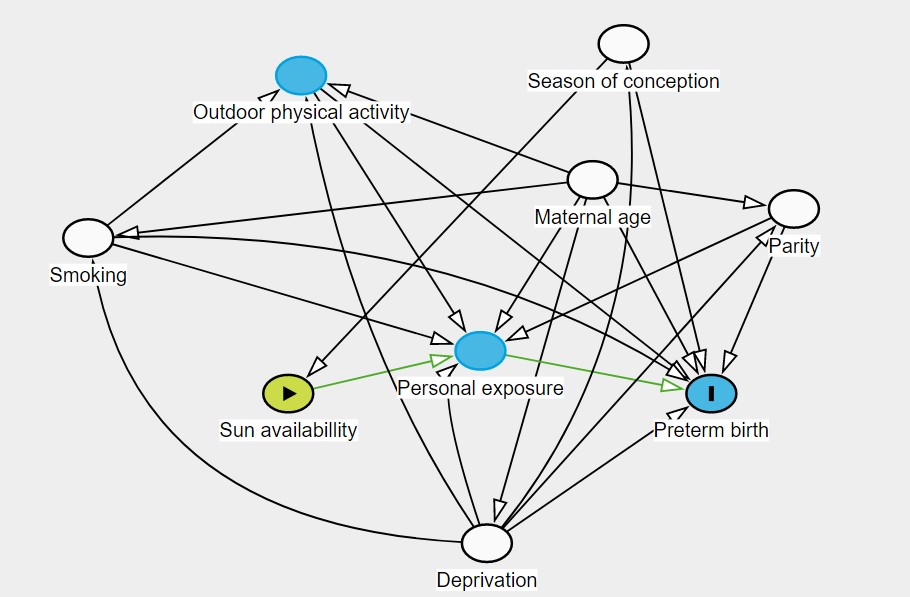

Supplement: Supplementary Figure 1 — Directional Acyclic Graph for main model. DAG shows the role of potential confounders in the relationship between environmental sunlight availability and gestational length of pregnancy. Adjusted and unadjusted variables are represented by white and blue circles respectively. This model indicates an unbiased pathway from sun availability to the outcome. [file Image_1.JPEG]

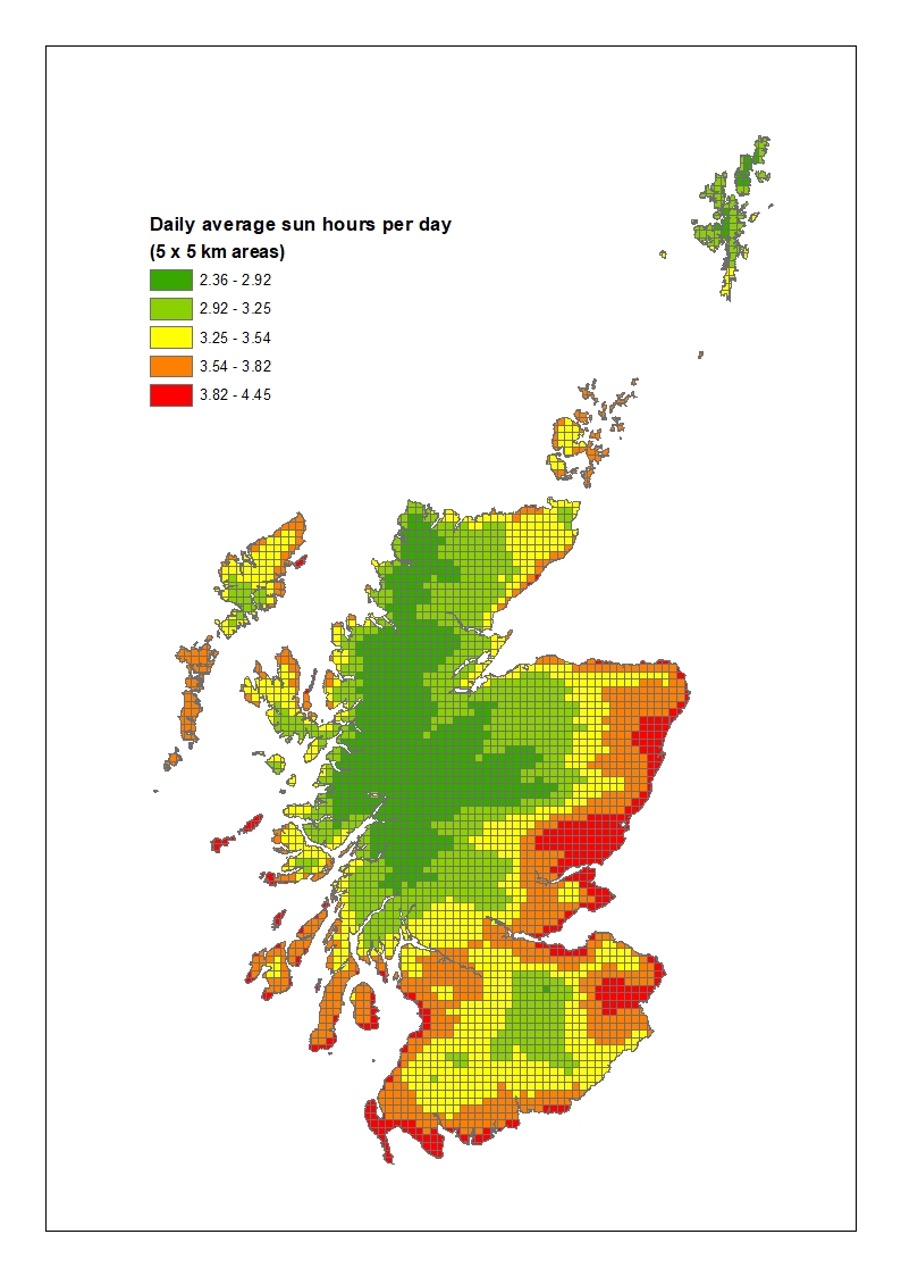

Supplement: Supplementary Figure 2 — Example of spatial variation in sun availability. Map shows average trimester 1 sun hours per day for pregnancies delivered in 2001 for each 5 ×5 km square in Scotland. [file Image_2.JPEG]
